# Developing a local service to improve the provision of palliative care to people who use substances

**DOI:** 10.1192/bjo.2021.526

**Published:** 2021-06-18

**Authors:** Vyasa Immadisetty, Natasha Palipane, Tracy Reed, Srirupa Gupta, Beverley Pickett, Fiona McDowall, Julia Thompson

**Affiliations:** 1 Essex Partnership University NHS Foundation Trust; 2 London School of Hygiene and Tropical Medicine, Farleigh Hospice; 3 Mid and South Essex NHS Foundation Trust; 4 East Suffolk and North Essex NHS Foundation Trust

## Abstract

**Aims:**

To develop a new service model that engages and improves the provision of palliative care to PWUS.

**Background:**

Although people who use substances (PWUS) continue to die prematurely compared to the general population, they are now more likely to die from chronic diseases rather than from drug-related deaths. Challenges to providing palliative care to PWUS include delayed care-seeking behaviours, complex drug interactions and lack of healthcare provider experience.

**Method:**

An informal factorial analysis elucidated population needs through: a review of local databases to estimate the prevalence of palliative need, a thematic review into the deaths of patients in specialist drug services and, a survey of health practitioners’ knowledge and attitudes. These informed the service development phase which involves three key components: 1. A systems approach to increasing patient identification, incorporating key multi-disciplinary stakeholders across hospital- and community-based care 2. Targeted training of healthcare providers and 3. Medicines management for symptom palliation amidst concurrent substance use (including substitution treatments).

**Result:**

The palliative needs of PWUS are under-identified: the local substance service was not partaking in the palliative referral pathway. Only 7% of a local hospice's annual caseload was recognised as having substance use problems. The care pathway was described as fragmented. Although >80% of surveyed palliative care practitioners had experienced caring for PWUS, confidence and knowledge around managing withdrawal, pain and opioid substitution therapies was poor.

**Conclusion:**

A new pathway is designed to identify PWUS and in their last year of life at key treatment points e.g., accident and emergency, ward-based care. The pathway will then streamline referrals to relevant specialist services depending on complexity of palliative/dependency need. Teaching resources and prescribing guidelines have been developed in collaboration with secondary care pain specialists.

